# Generation of recombinant MVA-norovirus: a comparison study of bacterial artificial chromosome- and marker-based systems

**DOI:** 10.1186/s12985-019-1212-y

**Published:** 2019-08-09

**Authors:** Franziska Kugler, Ingo Drexler, Ulrike Protzer, Dieter Hoffmann, Hassan Moeini

**Affiliations:** 1 Institute of Virology, Faculty of Medicine, Technische Universität München, Munich, Germany; 2 Institute for Virology, Universitätklinikum Düsseldorf, Heinrich Heine Universität, Düsseldorf, Germany

**Keywords:** Bacterial artificial chromosome, Recombinant MVA, Self-excising, Norovirus

## Abstract

**Background:**

Recombinant Modified Vaccinia Virus Ankara has been employed as a safe and potent viral vector vaccine against infectious diseases and cancer. We generated recMVAs encoding norovirus GII.4 genotype capsid protein by using a marker-based approach and a BAC-based system. In the marker-based approach, the capsid gene together with a reporter gene was introduced into the MVA genome in DF-1 cells. Several rounds of plaque purification were carried out to get rid of the WT-MVA. In the BAC-based approach, recMVA-BAC was produced by *en passant* recombineering in *E. coli*. Subsequently, the recMVAs were rescued in DF-1 cells using a helper rabbit fibroma virus. The BAC backbone and the helper virus were eliminated by passaging in DF-1 cells. Biochemical characteristics of the recMVAs were studied.

**Results:**

We found the purification of the rare spontaneous recombinants time-consuming in the marker-based system. In contrast, the BAC-based system rapidly inserted the gene of interest in *E. coli* by *en passant* recombineering before virion production in DF-1 cells. The elimination of the reporter gene was found to be faster and more efficient in the BAC-based approach. With Western blotting and electron microscopy, we could prove successful capsid protein expression and proper virus-assembly, respectively. The MVA-BAC produced higher recombinant virus titers and infected DF-1 cells more efficiently.

**Conclusions:**

Comparing both methods, we conclude that, in contrast to the tedious and time-consuming traditional method, the MVA-BAC system allows us to quickly generate high titer recMVAs.

## Background

Norovirus is highly infectious and can be serious to individuals with underlying conditions, the elderly, young children and immunocompromised patients. GII.4 genotype evolves faster than other genotypes and global pandemic outbreaks often correlate to the emergence of new GII.4 variants [1–3]. Major capsid protein (VP1) is responsible for virus attachment to the histo-blood group antigen [4]. The capsid protein is divided into shell (S) domain and a protruding (P) domain linked together by a hinge of eight amino acids [4, 5]. The aim of this study was to generate a recMVA expressing norovirus (NoV) capsid protein of GII.4 genotype. We plan to apply the constructs as vaccine candidates against this most prevalent gastroenteritis virus.

Modified Vaccinia Ankara (MVA) is a highly attenuated strain of vaccinia virus derived by extensive serial passages in chicken embryo fibroblasts (CEF) [6]. MVA can be produced in large scale in chicken cell lines under bio safety level 1. Temporal high expression of MVA-delivered antigens can modulate antigen-specific immune responses, making it a suitable vaccine carrier [6–9]. We followed a traditional marker-based method (as reviewed by Drexler et al. [7]) and a BAC-based approach as described by Dai [10] and Cottingham et al. [11] for generation of recMVA-NoV constructs. Both techniques produced recombinant MVA delivering the desired antigen.

**Fig. 1:**
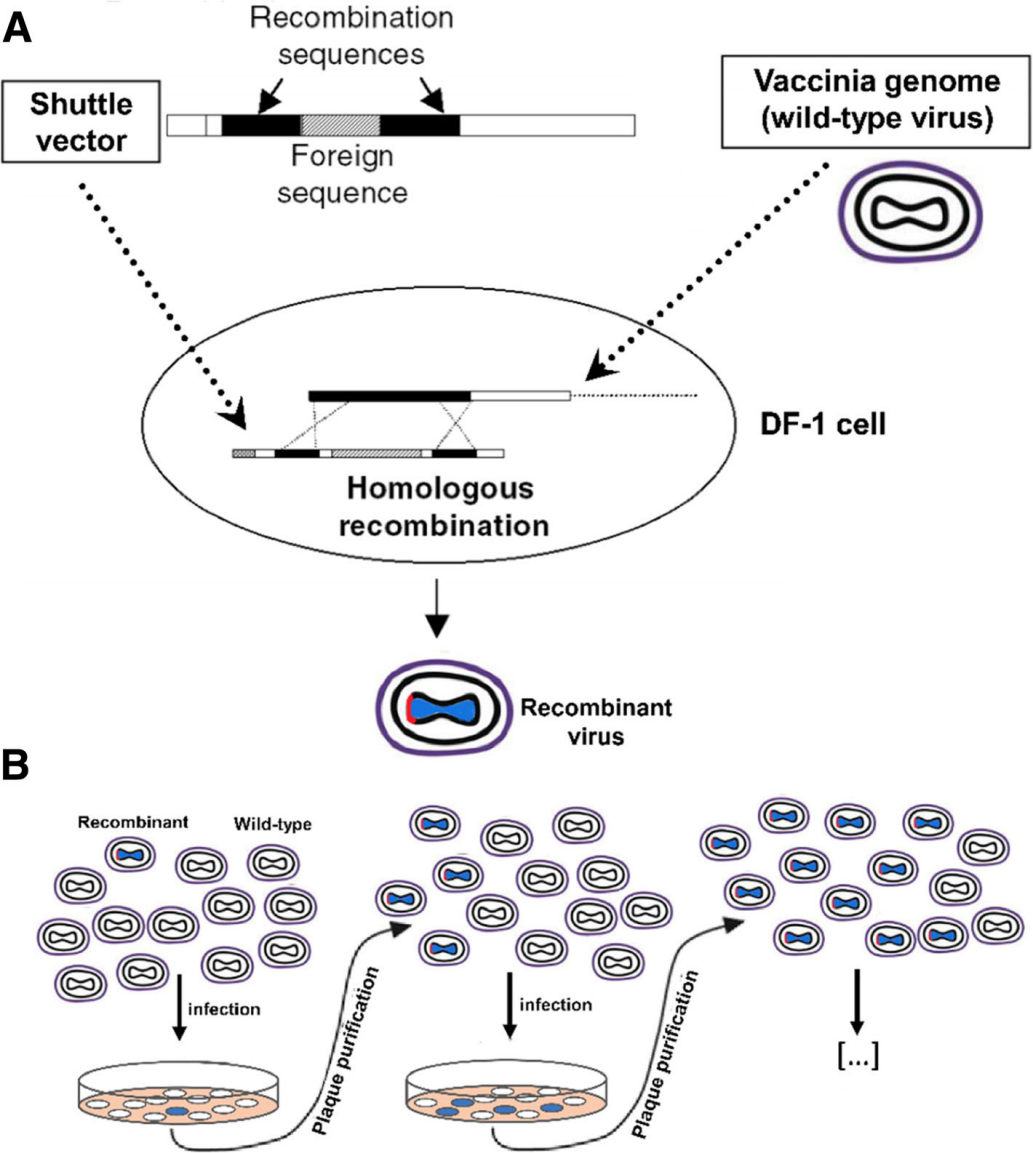
Schematic overview of traditional marker-based method for generation of recMVA. **a** Construction of a recombinant vaccinia virus by homologous recombination in DF-1 cells infected with WT MVA. **b** Plaque purification of recMVA. Adapted from Nagel [13] and Rocha [14] with modifications

In the marker-based approach, a shuttle vector harboring a fluorescence reporter gene, the gene of interest (GOI) is inserted downstream of the modified H5 (mH5) promoter [12]. In wild-type MVA-infected cells, the gene of interest together with the reporter gene is introduced into the viral genome by homologous recombination (Fig. 1). Plaque purification is carried out to eventually get rid of the WT-MVA. To this end, fluorescent plaques are picked and tested for the presence of WT-MVA by PCR. As homologous recombination is not 100% efficient, both of WT- and rec-MVA viral particles are released in the supernatant (Fig. 1b) and therefore, several rounds of plaque purification is required to eradicate the WT virus. The reporter gene will be eliminated from the MVA genomes gradually, as it is flanked by homologous regions that can recombine during replication. Selection for infected cells is difficult, as DF-1 cells do not form morphologic distinct plaques.

**Fig. 2:**
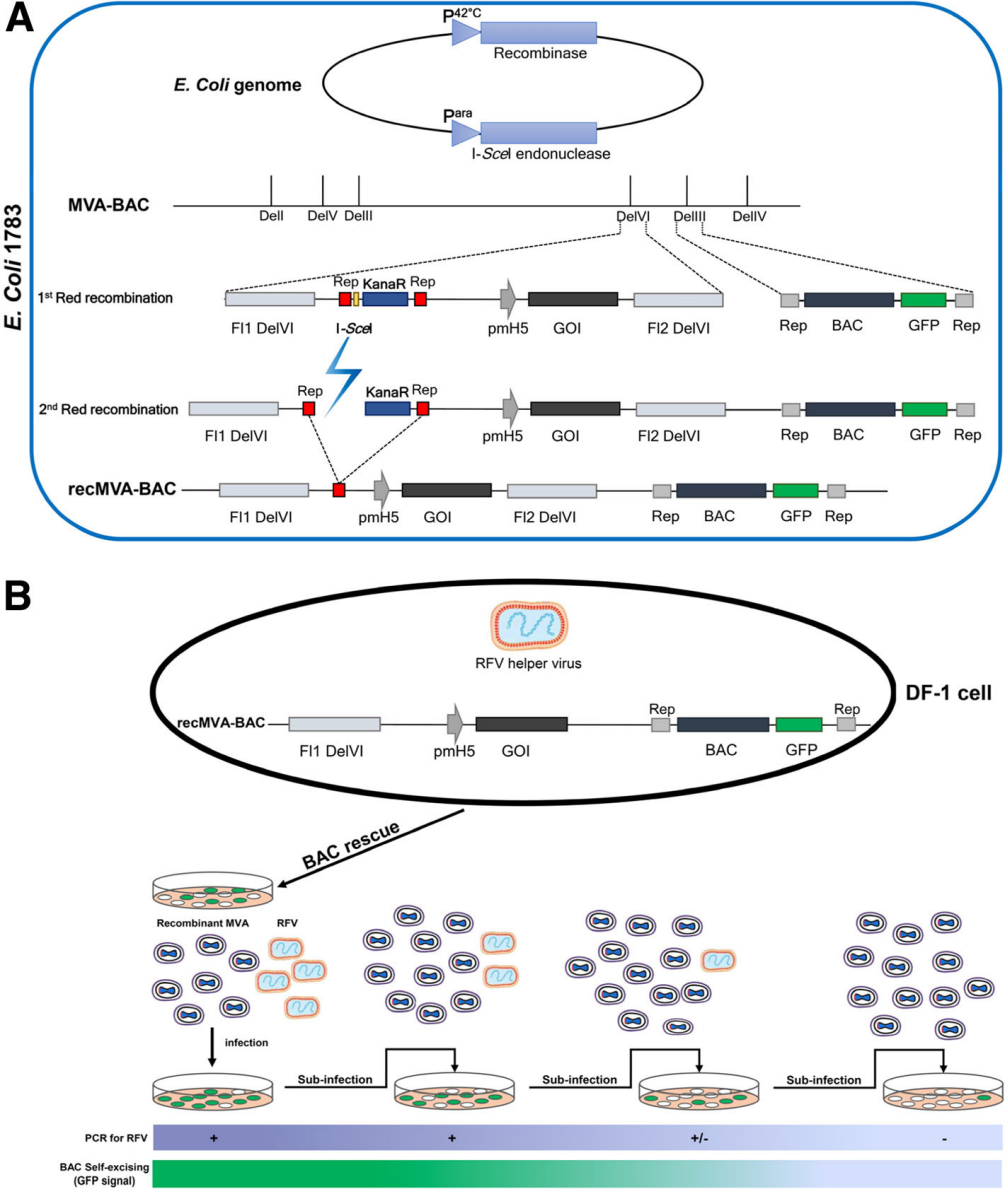
Schematic overview of MVA-BAC technique for generation of recMVA. **a** In *E. coli* GS1783, following induction at 42 °C, the gene of interest together with I-*Sce*I-Kan fragment is inserted into the *Del*VI site of the MVA-BAC by homologous recombination. Co-integrated Kan cassette (aphAI) is removed by induction of I-*Sce*I production by arabinose followed by second Red recombination **b** In DF-1 cells, recMVAs are rescued in present of a helper virus and the BAC backbone including GFP cassette is removed spontaneously upon passaging

In the BAC-based system, recMVA is produced by *en passant* recombineering as previously described by Dai [10, 11]. Red-mediated modification is performed in the *E. coli* strain GS1783 containing a temperature-dependent expression cassette for the recombination proteins and an L-arabinose-inducible I-*Sec*I gene. In the first step, as shown in Fig. 2a, the gene of interest is introduced into a shuttle vector which is equipped with homologous regions to the *Del*VI of the MVA genome in the MVA-BAC plasmid. After heat induction, the Red recombinase promotes the insertion of our gene of interest along with the Kanamycin (Kan) resistance cassette and the I-*Sce*I restriction site in the MVA genome. Positive clones are selected on agar plate supplemented with kanamycin and after confirmation of the insertion, selected BAC plasmids are subjected to the 2nd Red recombination. I-*Sce*I endonuclease induction is triggered by arabinose to eliminate Kan resistance gene. Subsequently, to rescue recombinant progeny virus, recMVA-BACs are introduced into DF-1 cells, as presented in Fig. 2b. In this step, a helper virus which provides the initial transcription machinery for triggering recMVA replication, is required. As the BAC construct contains a GFP cassette, successfully infected cells can be monitored by fluorescence microscopy. In a final step, by sub-infection of DF-1 cells with the recMVAs and due to self-excising property of the system, BAC backbone containing GFP cassette is eliminated. The helper virus will also be cleared by passaging steps in DF-1. These can be confirmed by PCR using specific primers for *gfp* gene and helper virus genome.

## Results

### Generation of recombinant MVA by homologous recombination in DF-1 cells

**Fig. 3:**
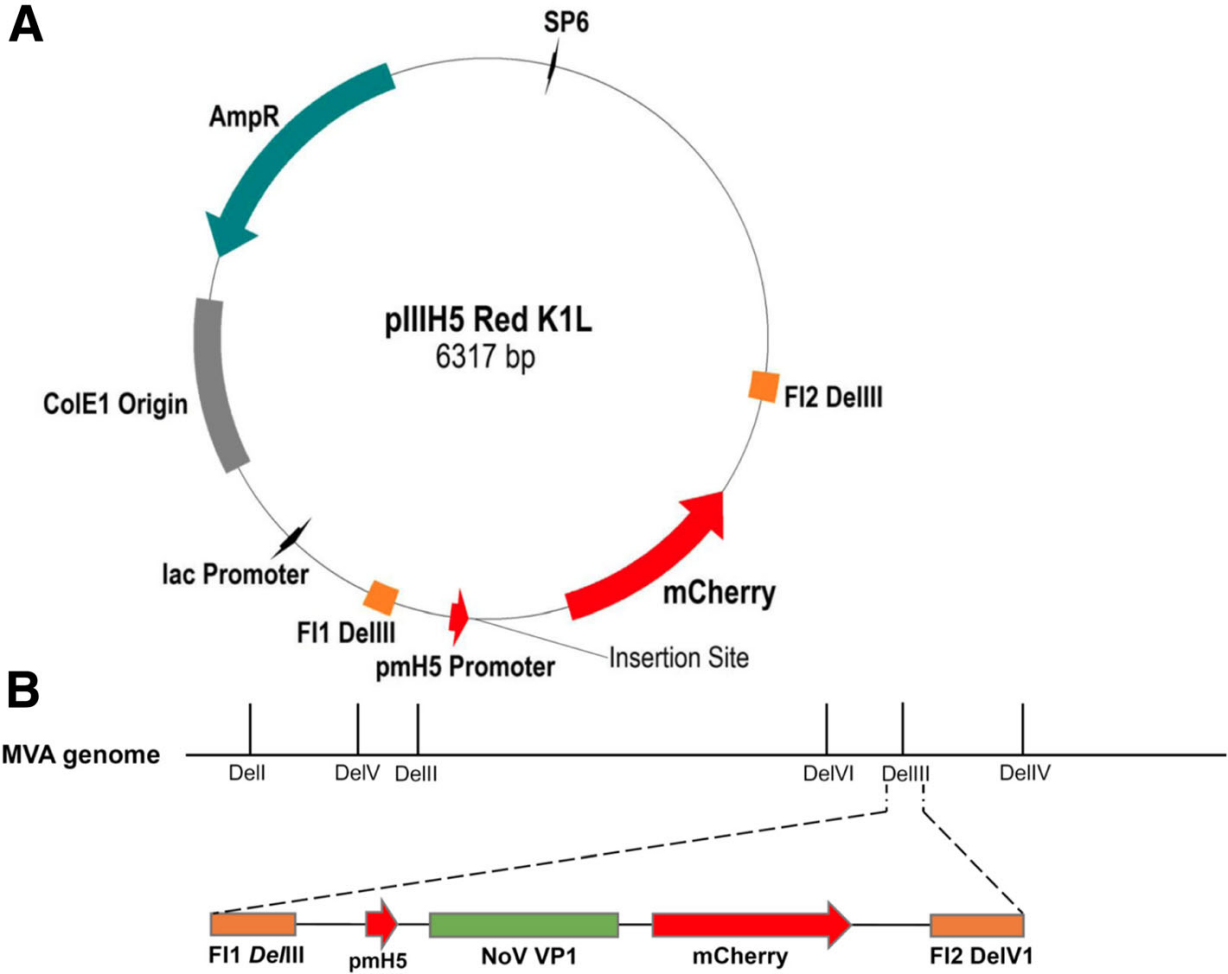
**a** Schematic map of shuttle vector pIIIH5 Red K1L. **b** Targeting expression cassette into MVA *Del*III region by homologous recombination of adjacent flank sequences FI1 *Del*III and FI2 *Del*III

NoV GII.4 capsid gene was inserted into the *BamH*I site of the pIIIPH5 Red K1L plasmid (Fig. 3b) to construct a shuttle vector, namely pIIIH5-GII4VP1. After transformation into *E. coli* DH5, insertion was verified by PCR and restriction enzyme digestion analysis. The integrity of the inserts was confirmed by sequencing.

**Fig. 4:**
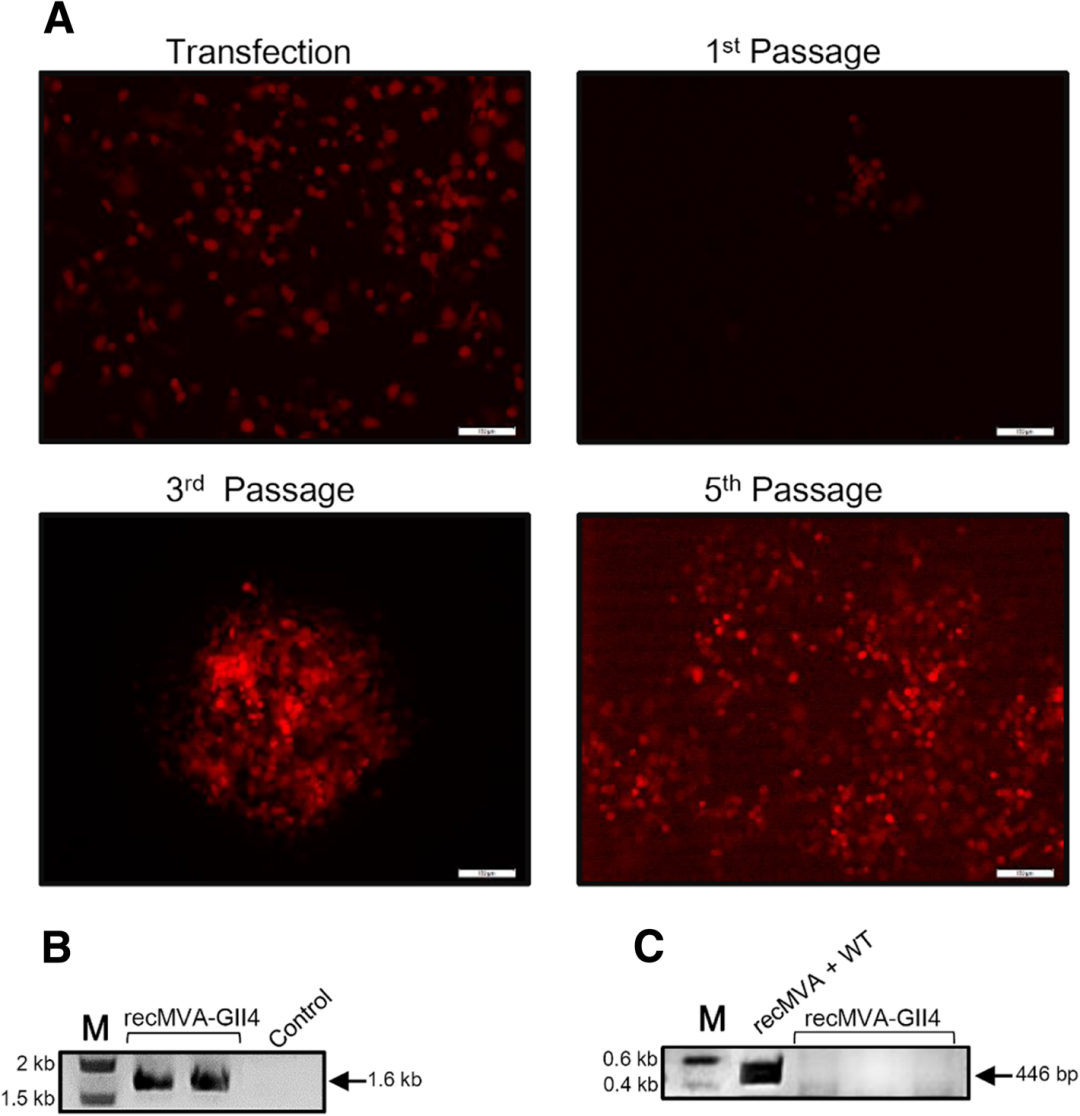
Generation of recMVA-NoV using marker-based approach. **a** After transfection of the pIIIH5-GII4VP1 into MVA-infected DF-1 cells, mCherry-expressing cells producing recMVAs were picked and plaque purification was carried out. After some rounds of plague picking, a significant increase in the signal was observed. Scale: 100 μm. **b** Confirmation of gene insertion into the MVA genome by PCR. After extraction of viral DNA from infected DF-1 cells, insertion of the NoV capsid gene was checked by amplifying the *Del*III cassette with gene-specific primers. An amplified fragment of 1.6 Kb was expected for the GII.4. DF-1 cells infected with WT-MVA were used as negative control for PCR. **c** Confirmation of WT-MVA clearance. The presence of the WT-MVA was checked by amplifying *Del*III cassette where a PCR product of 446 bp was expected

The shuttle vector was introduced into DF-1 cells infected with the WT-MVA. Forty-eight hours post-transfection, the cells were checked for expression of mCherry. As shown in Fig. 4a, in the 1st passage, we could detect red plaques producing recMVA. Insertion of NoV capsid gene was confirmed by PCR (Fig. 4b). In each step, the presence of the WT MVA was checked by PCR. After several rounds of plaque purification, WT-MVA DNA became undetectable for the recMVA-GII.4 (Fig. 4c).

### Generation of recombinant MVA-BAC

**Fig. 5:**
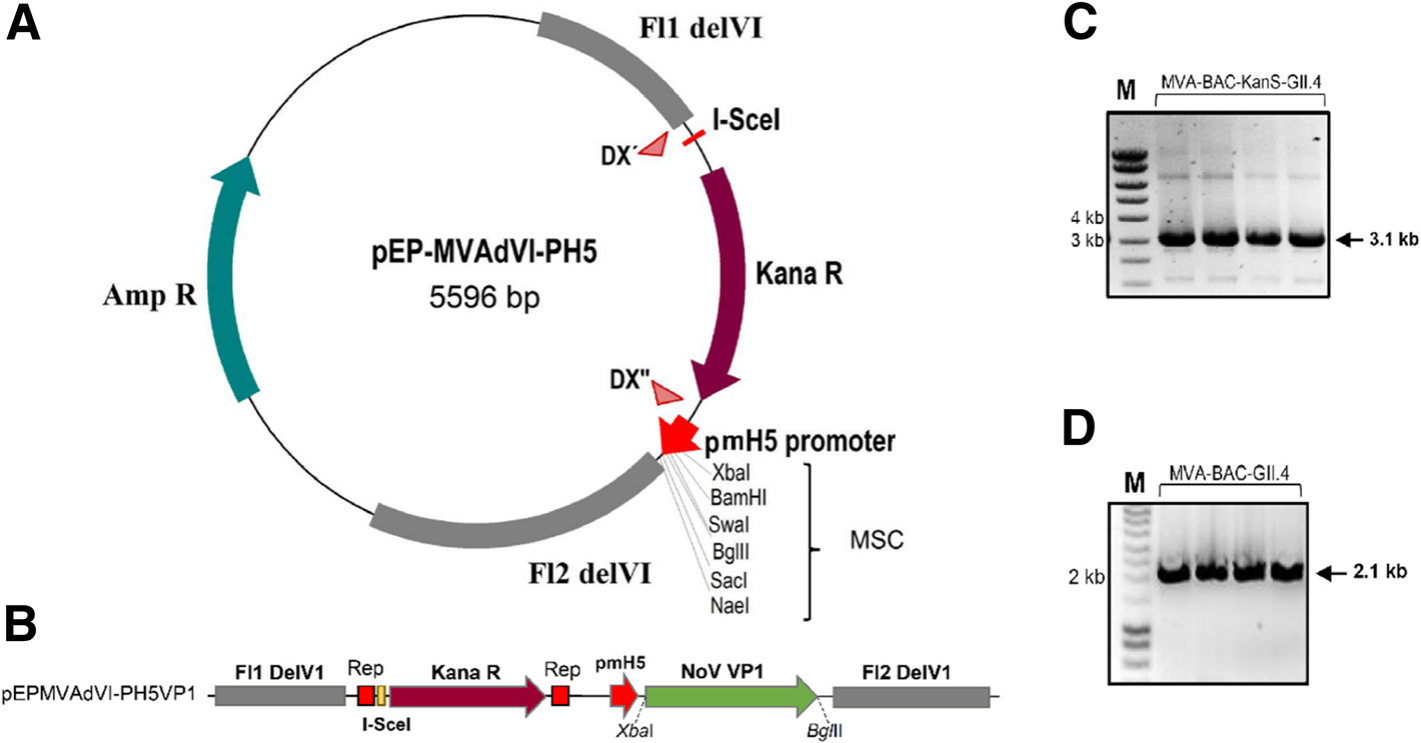
Generation of recMVA-NoV BAC plasmid. **a** Schematic map of the shuttle vector pEP-MVAdVI-PH5. **b** Linear map of insertion region in the recombinant pEPMVAdVI-PH5VP1. The recombinant plasmid was constructed by insertion of the NoV GII.4 capsid gene VP1 into the shuttle vector downstream of the viral promoter PmH5. **c** Confirmation of first Red recombination. After first recombination in *E. coli 1783*, insertion of the I-*Sec*I-Kan-pH 5/VP1 fragment into MVA-BAC genome was confirmed by PCR (expected size: 3.1 kb). **d** Confirmation of resolution of co-integrated Kan cassette by PCR using *Del*VI-specific primers (expected size: 2.1 kb)

Recombinant shuttle vectors carrying NoV capsid gene were generated by insertion of the GII.4 VP1 gene into the *Bgl*II and *Xba*I sites of the pEPMVAdVI PH5 plasmid downstream of the PmH5 promoter (Fig. 5b). After transformation into *E. coli* competent cells, correct clones were identified by RE digestion and PCR using gene-specific and *Del*VI primers. Potential correct clones were checked for integrity of the inserts by sequencing. To generate recombinant MVA-BAC, the transgene cassette along with the Kan cassette, I-*Sec*I sequence and *Del*VI flanking sequences were amplified from the shuttle vectors and introduced into the GS1783 *E. coli* competent cells carrying the MVA/BAC. As shown in Fig. 5c, PCR amplification confirmed the insertion of the I-*Sec*I-Kan-pH 5/NoVGII4 fragment into the MVA genome in the BAC plasmid. The KanS-cassette was removed by arabinose induction of endonuclease, I-*Sce*I followed by induction of the second Red recombination at 42 °C in the GS1783 *E. coli* cells. Correct clones were confirmed by PCR using specific *Del*VI primers (Fig. 5d). Restriction fragment analysis using *BamH*I and *EcoR*I also confirmed correct insertion of the VP1 capsid gene (Data not shown).

**Fig. 6:**
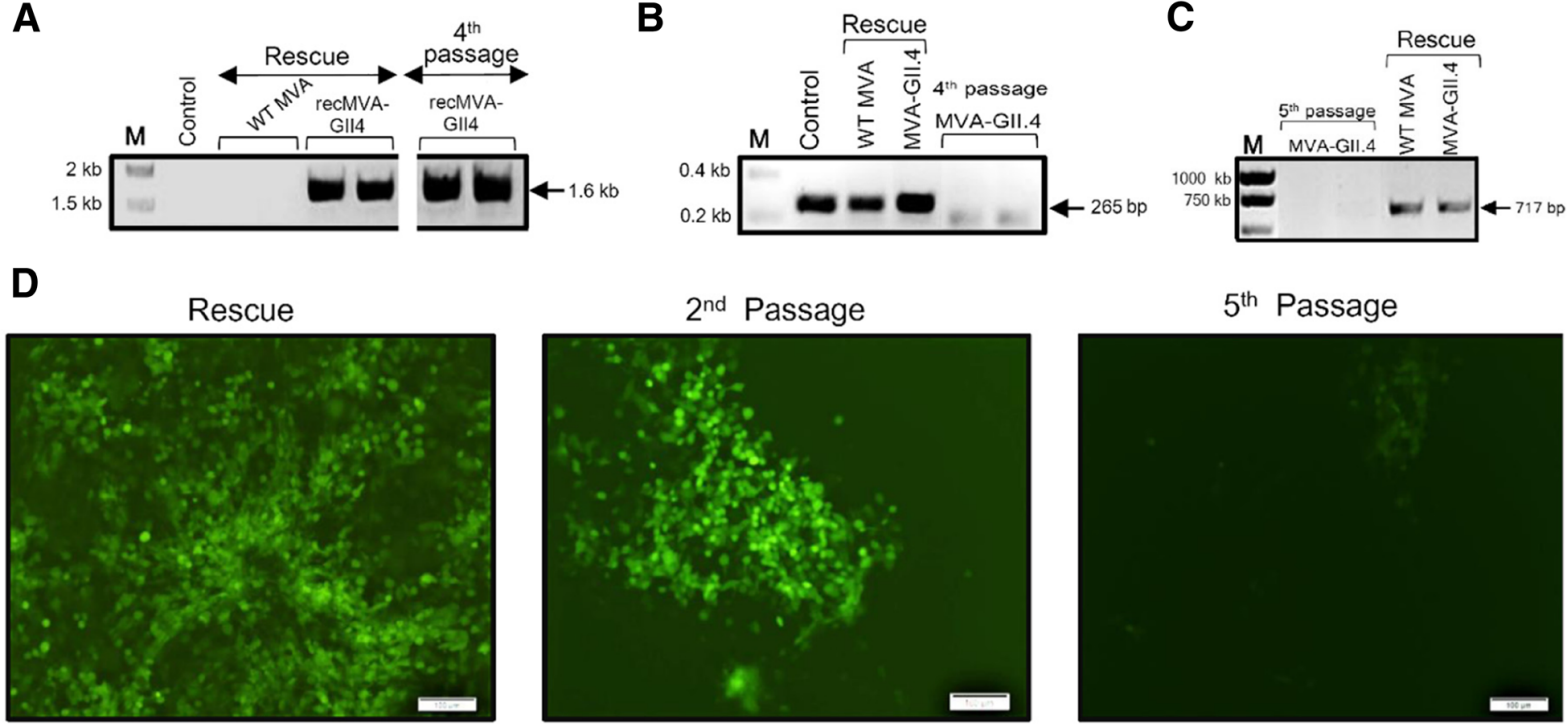
Rescue of recMVA-NoV from recBAC plasmid in DF-1 cells. **a** Confirmation of gene insertion into the MVA genome by PCR. Insertion of the capsid gene was checked by amplifying the *Del*VI cassette using *Del*VI primers for the WT (expected size: 498 bp) and gene-specific primers for the recMVA-BAC-GII.4 (expected size: 1.6 kb). Uninfected DF-1 cells were used as negative control. **b** Confirmation of RFV clearance by PCR using RFV-specific primers with an expected size of 265 bp. RFV-related band was detected in the first rescue sample; it was not detectable from the 4th passage. Purified RFV was used as positive control. **c** and **d** BAC self-excision: After transfection of the shuttle vector into RFV-infected DF-1 cells, the BAC backbone and the GFP cassette were spontaneously lost by passaging: (C) Confirmation of removal of BAC backbone by PCR using specific primers for *gfp* gene. (D) significant reduction in GFP population indicating the loss of BAC cassette from the recMVA genome. Scale: 100 μm

The MVA-BAC-GII4VP1 plasmid was transfected into DF-1 cells after infection of the cells with the helper virus RFV. After 2–3 days of incubation at 37 °C, the cells were harvested and the recombinant viruses were used to infect new DF-1 cells. PCR analysis revealed successful integration of the capsid genes (Fig. 6a). Clearance of the helper virus from the recMVA-producing cells was confirmed, after four rounds of passaging (Fig. 6b). Due to self-excising property of the MVA-BAC, the BAC-GFP cassette was expected to be eliminated from the MVA genome by an inverse-oriented sequence replication in the *Del*III of the genome [15]. As shown in Fig. 6d, after five rounds of recMVA passaging we observed a significant reduction in GFP population indicating the loss of BAC cassette from the *Del*III locus of the recMVA genome. Successful removal of BAC-GFP marker was assured by PCR using specific primers for the *gfp* gene, as shown in Fig. 6c.

### Biochemical characterization of the recMVAs

**Fig. 7:**
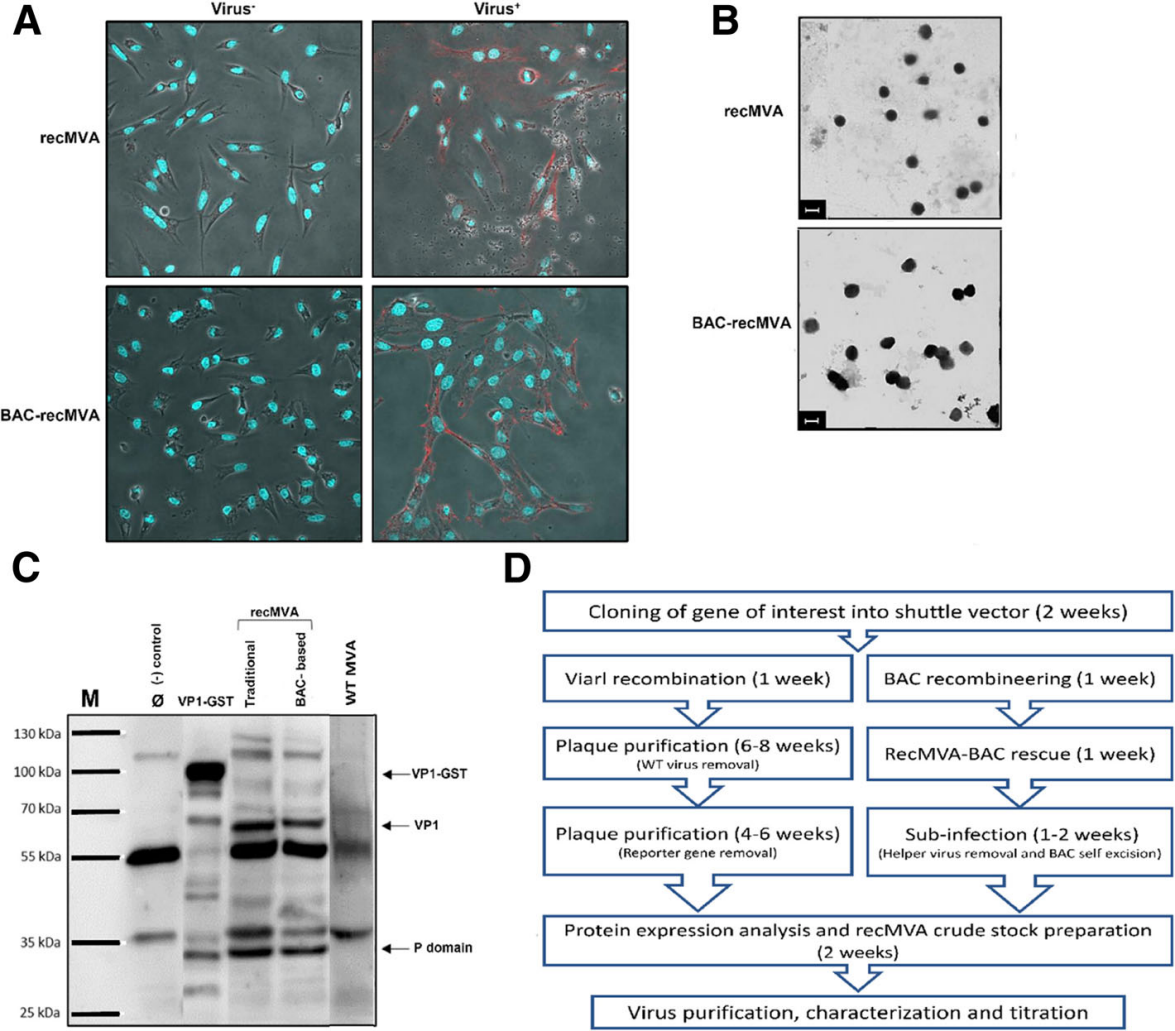
Biochemical characterization of recMVAs. **a** Binding assay: Control cells are depicted on the left side. Virus binding to DF-1 cells was visualized by staining with anti-Vaccinia virus antibody. Top: recMVAs derived by the marker-based method; Bottom: BAC-recMVAs. **b** Electron microscopy with negative staining. Scale: 300 nm. **c** Expression of NoV VP1 in DF-1 cells infected with recMVAs. Immunoblotting was carried out using human serum from GII-infected patients as primary- and anti-human IgG as secondary antibody. VP1 protein (58 kDa) was detected in both recMVAs. Non-infected- and WT-MVA-infected cells were used as negative controls. Recombinantly expressed GST-tagged GII.4VP1 was used as positive control. **d** Schematic workflow for recMVA generation using traditional approach (left) and MVA-BAC system (right)

Binding assay clearly showed binding of the recMVAs on the cell surface, as shown in Fig. 7a. Propagated recMVAs were purified from cell suspension and after negative staining were visualized by EM where enveloped, brick-shaped MVA virions with a size of approximately 300 × 250 nm [16] were detected (Fig. 7b). Successful expression of NoV capsid protein in the recMVA-producing DF-1 cells was confirmed by Western blotting, where we could clearly detect a 58 kDa VP1 band for both constructs (Fig. 7c). A 32 kDa protein band, the P domain size, was also detected. According to a previous study [17], soluble VP1 is susceptible to in vivo proteolytic trypsin cleavage at aa residue 227, leaving a 32 kDa protein. The cleavage site is thought to be buried in assembled virions, while it becomes exposed when the particles assemble to multimers or unfold. The absence of the S domain is thought to be due to intercellular degradation [15, 18]. The bands we assume as P particles or multimers were not present in the uninfected control. Recombinant GST-tagged GII.4 VP1 protein expressed in *E. coli* and non-infected cells were used as positive and negative controls, respectively. Plaque-forming units (PFU) were determined for the virus stocks in DF-1 cells infected in duplicates with the respective virus in 10-fold serial dilutions. Virus titers of 4.1 × 10^8^- and 1.7 × 10^9^ PFU/ml were measured for the marker-based- and the BAC-based systems, respectively.

## Discussion

The highly attenuated vaccinia virus, MVA, has been established as a safe and potent viral vector for the development of recombinant vaccines [7, 19–21]. MVA-based vaccines have been shown to induce a robust T cell response to the transgene [16, 22, 23]. A short burst of antigen expression following immunization makes it also suited for B cell boosting [24]. In the present study, we generated recMVA vectors encoding norovirus GII.4 capsid protein to be used as B- and T- cell booster in a prime-boost vaccination regime. In this work, beside the available marker-based approach, a BAC-based system was applied for generation of recombinant MVA vectors. The marker-based approach relies upon homologous recombination with a shuttle vector in virus-infected cells. We used pIIIH5 Red K1L as shuttle vector where the NoV GII.4 capsid gene was inserted downstream of the modified H5 promoter PmH5 which contains both native early and late vaccinia promoter regions [25]. PmH5 is a strong vaccinia virus promoter that overexpresses the respective antigens in a high dose, during the early and late phase of virus infection [12]. It has been shown that antigen driven by PmH5 elicited dramatically a higher immunogenicity than those by other vaccinia virus promoters [26, 27]. In addition, mH5 promoter improves stability of insert genes during extended passages when compared with some other strong promoters such as pSyn I or II [12]. The transgene was designed so that it can recombine into the *Del*III site of the MVA genome by homologous flanks adjacent to the gene of interest. Forty-eight hours after introducing the shuttle vectors into DF-1 cells, recMVA-producer cells were recognized by mCherry expression. In early passages, plaque picking was tedious, as only a few cells were infected and a high number of plaques had to be isolated for the second round of purification. After several rounds of plaque purification from infected cells with highly diluted virus-titers we managed to eliminate wild type MVA. The reporter gene mCherry is removed by spontaneous homologous recombination of two flanking sequences. Therefore, after some rounds of plaque purification we expected a decrease in fluorescence signal with an increase in the recMVA yield. Eliminating the reporter gene is particularly important when the potential vaccine is required for clinical applications. The production of this markerless construct brought up another drawback as MVA does not form morphologically distinct plaques in DF-1 cells. Hence, picking of mCherry-negative plaques issued another challenge. We could not eliminate the mCherry reporter marker after 10 rounds of passaging (data not shown). Even though the marker-based generation of MVA recombinants is robust, purification of the rare spontaneous recombinants is time-consuming and cumbersome.

In the BAC-based system, a self-excising variant of MVA-BAC was applied in which the BAC cassette harboring the selection marker is spontaneously removed from derived recombinant viruses. The major advantage compared to the marker-based attempt is the utilization of *E. coli* as a production platform. In this way, positive clones harboring the expression cassette can easily be identified by their Kanamycin resistance. In contrast, the only way to determine positive clones in the marker-based system is mCherry expression in infected DF-1 cells. Hence, the MVA-BAC system guarantees the successful incorporation of the gene of interest at a stage much ahead of the actual production of virions in DF-1 cells. GFP-expression facilitated evaluation of correct transfection of DF-1 cells significantly and, importantly, the cassette was lost after few rounds of sub-passaging. Compared to the marker-based approach, the elimination of the marker gene scar was found to be vastly superior in the BAC-system. In contrast to the marker-based methods, where several rounds of plaque purification are required for elimination of the WT virus from the culture, in MVA-BAC approach RFV, providing initial transcriptional products for virus replication, was eliminated very fast, after two rounds of passaging. RFV infection in DF-1 cells is abortive as the virus is unable to replicate in these cells.

After successful production of recMVAs, stable integration of the capsid gene into the genome was confirmed. Successful expression of the capsid protein was then confirmed by visible VP1 band in Western blot for the recombinants. With electron microscopy, we could see the expected round 300 × 250 nm viral particles [16]. We also visualized recMVAs by a binding assay. Attachment of the recombinant MVA particles on the DF-1 cell surface was clearly visible; even though our subjective impression suggested a higher binding rate for the recMVA-BACs.

Not only high virus titer was measured with recMVA-BACs, but also infection of new DF-1 cells was more efficient than with the recMVAs obtained by the marker-based method. We therefore hypothesize that the MVA-BAC platform provides more functional viral particles. This might be due to the location of transgene insertion. The marker-based approach uses *Del*III, whereas MVA-BAC aims for *Del*VI. *Del*III has occurred in earlier passaging stages during MVA generation whereas *Del*VI has occurred 190 passages later. Interestingly, these deletion sites are in close proximity within the MVA genome. It still is not known what effects the deletion sites have on attenuation [28, 29]. However, these factors may contribute to the anticipated different protein expression levels as well as infection- and amplification properties of the virus.

## Conclusions

In this study, we successfully constructed recombinant MVA expressing norovirus capsid protein of GII.4 genotype by two different approaches. We conclude that the MVA-BAC system quickly produces high amounts of functional recombinant viral particles. The generation of the expression cassette, manipulate the MVA genome and removal of the selective marker resistance gene is done in *E. coli*, which simplifies handling and generation of recombinant MVAs.

Although MVA is known to be a potent boosting vector, it has limitations for priming immunostimulatory responses [20]. MVA can induce CD8^+^ and accompanying CD4^+^ T cell response and a short burst of antigen expression also boosts B cells [16, 19, 21]. Therefore we suggest using recMVAs as booster for B- and T- cell responses in combination with protein-, DNA- and/or mRNA vaccines.

## Methods

### Plasmids, bacterial strains, cells and viruses

DF-1 chicken fibroblast cells were used for MVA replication. Wild-type MVA [30] was obtained from the Institute of Infectious Diseases and Zoonosis, University of Munich LMU. MVA-BAC plasmid and helper Rabbit Fibroma Virus (RFV) was obtained from the Institute of Virology, Universitätklinikum Düsseldorf. *E. coli* GS1783 was used for generation of recMVA-BAC plasmid. Cloning plasmid pEX-K4-NoVGII.4 was used as a template for amplification of the NoV capsid gene. The pIIIPH5-RedK1L plasmid (Fig. 3a) was used as a shuttle vector for generation of recMVA by the marker-based approach. It contains an expression unit under control of a PmH5 promoter [12]. Homologous recombination with the non-essential *Del*III of MVA is directed by two stretches of genomic MVA sequences flanking the foreign gene and the mCherry cassette. The pEP-MVAdVI-PH5 plasmid containing two homologous sequences to the *Del*VI of the MVA genome was used as a shuttle vector for generation of recMVA-BAC. It contains MVA DNA homologous flanking sequences (FL1/2) adjusted to the MVA deletion VI (*Del*VI) region. The plasmid encodes for the ampicillin resistance gene as well as for the kanamycin resistance gene aphAI. There is an 18-bp recognition site of the endonuclease I-*Sce*I upstream the Kan cassette. I-*Sce*I generates a double-strand break at the recognition site without cleavage of human, bacterial or viral DNA [31]. Adjacent to the I-*Sce*I sequence and the Kan cassette, the 51 bp homologous sequences DX´ and DX´´ ensure the elimination of the marker gene by homologous recombination.

## Generation of recMVA in MVA-infected DF-1 cells

### Construction of MVA shuttle vector

The full length of NoV GII.4 capsid gene was amplified from the pEX-K4-GII.4 plasmid using a pair of specific primers listed in Table 1, and inserted into the *BamH*I site of the shuttle vector pIIIH5-RedK1L downstream of the PmH5 promoter using In-Fusion cloning kit (Takara Bio, USA). After transformation into Top10 *E. coli* competent cells, positive clones were screened on LB-Amp (100 μg/ml) plates. Recombinant plasmids were checked for gene insertion by restriction enzyme (RE) digestion. Integrity and correct orientation of the insert were verified by double-stranded sequencing. The constructs were purified in large-scale using the Plasmid *Plus* Midi Kit (Qiagen, Germany).

**Table 1:** List of primers used in this study

Primer	Sequence 5′ → 3´	Description
Kan control	F: CGTACTCCTGATGATGCATG R: ATTCGTGATTGCGCCTGAGC	For control PCR/sequencing after first recombination into MVA-BAC
MVA *Del*III	F: GATGAGTGTAGATGCTGTTATTTTG R: GCAGCTAAAAGAATAATGGAATTG	To check the presence of WT and gene insertion after plaque picking
MVA *Del*VI	F: CTCCGCATCTAGTTGATATTCCAACCTCTT R: CCTGGACATTTAGTTTGAGTGTTCCTGAAT	For first homologous recombination into the MVA-BAC by *En-passant*
pEPH5-GII4	F: CATAAATAAGGTTGACTCTAGAGCCACCATGAAGATGGCCTC R: ACGTAGAGCTCTTAAGGAATTCTTATACGGCTCGTCTTCTACCT	For cloning of NoV GII.4 into pEP-MVAdVI-PH5 plasmid
pIIIH5-GII.4	F: TAAGGTTGACTCTAGAGCTAGCGCCACCATGAAGATGGCCTC R: CGGCCGCGTTTAAACCTCGAGTTATACGGCTCGTCTTCTACCT	For cloning of NoV GII.4 into the pIIIH5 shuttle vector
RFV	F: AAAGATGCGTACATTGGACCC R: GTTCGAGACTAGAAAAGCGCC	To check the presence of helper virus RFV in the transfect cells with recMVA-BAC

### Generation of recMVA by homologous recombination

At confluency of approximately 90%, DF-1 cells were infected with WT-MVA at MOI 0.05 for 1.5 h at 37 °C. The cells were then transfected with the pIIIH5-RedK1L-GII.4VP1 plasmid using Lipofectamin® 2000 transfection reagent (Thermo Scientific, USA) as described by Moeini et al. [32]. Forty-eight-hour post-transfection, the cells were harvested by scraping and centrifugation at 4000 rpm, 4 °C for 10 min. The cells were resuspended in 1 ml media and viral particles were released from the cells by 3 times freeze-thawing. Cell debris was pelleted at 14,000 rpm for 10 min, and 200 μl of the virus-containing supernatant was used to infect new DF-1 cells in 6-well plates in a serial dilution (10^− 1^–10^− 6^). Two days post-infection, the cells were checked under fluorescence microscope for the expression of the reporter gene, mCherry. Red plaques were picked and used to infect new DF-1 cells for further purification of the mCherry-expressing plaques. Plaque picking was carried out for several rounds to get rid of the WT-MVA. In each step of plaque purification, the cells were checked for the presence of the WT-MVA by PCR using *Del*III-specific primers listed in Table 1.

### Generation of recMVA by BAC-based system

#### Construction of MVA shuttle vector

Norovirus GII.4 capsid gene was amplified from the pEX-K4GII.4 plasmid using a pair of specific primers listed in Table 1 and inserted into the *Xba*I and *Bgl*II sites of the shuttle vector pEP-MVAdVI-pH 5 using In-Fusion cloning kit. After transformation into *E. coli* competent cells, positive clones were screened on LB-Amp agar; plasmids were extracted and subsequently gene insertion was confirmed by PCR and RE digestion. Finally, the integrity of the gene insert was confirmed by sequencing.

#### Insertion of the VP1 gene into the MVA-BAC genome

*E. coli* GS1783 carrying MVA-BAC (GS1783/MVA) was grown overnight at 32 °C in LB broth supplemented with 30 μg/ml chloramphenicol (Cam); electrocompetent cells were prepared as previously described [33]. The GII4 VP1 gene along with the KanS fragment (including Kan cassette and I-*Sce*I restriction site) was amplified from the pEP-MVAdVI-pH 5-GII4VP1 plasmid using MVA *Del*VI primers listed in Table 1. The PCR products were treated with *Dpn*I, purified and introduced into the electrocompetent *E. coli* GS1783/MVA cells for the first Red recombination. Briefly, in a pre-chilled electroporation cuvette, 100 ng of the PCR products was added to 50 μl of the electro-competent cells and electro-transformation was carried out at 15 kV/cm, 25 μF, 200 Ω using the Gene Pulser Xcell Electroporation device (Bio-Rad, USA). The cells were immediately mixed with 1 ml LB broth followed by 1 h incubation at 32 °C. Positive clones were screened on LB/cam agar after 24 h incubation at 32 °C. Recombinant MVA-BAC plasmids were extracted using the GeneJet Plasmid Miniprep Kit (Thermo Scientific, USA) and gene insertion was confirmed by PCR using the *Del*VI primers.

To remove the co-integrated Kan cassette from the inserts, in 1 ml LB-Cam broth medium, *E. coli* cells carrying recombinant MVA-BAC were inoculated at 32 °C and 220 rpm for 2 h until the culture became cloudy. Thereafter, to induce the production of I-*Sce*I, 1 ml pre-warmed LB-Cam medium + 2% arabinose was added to the culture followed by 1 h incubation at 32 °C and 220 rpm. The cells were then heat-shocked at 42 °C in a shaker water bath for 30 min to induce Red recombinase. After 2–3 h incubation at 32 °C in a shaker incubator, the cells were screened on a LB-Cam agar plate supplemented with 1% arabinose. After 2 days of incubation at 32 °C, positive clones were tested for the resolution of the co-integrated cassette by PCR.

#### MVA BAC-rescue in DF-1 cells

In 6-well plates, at confluency of 80–90%, DF-1 cells were transfected with the recMVA- BAC GII4VP1 plasmid using Lipofectamin® 2000 transfection reagent. For reactivation of MVA, after 3 h incubation at 37 °C, the cells were infected with the helper virus RFV at MOI 1 with gentle rotation [34]. One hour later, the wells were filled up with RPMI medium supplemented with 10% FCS. After 24–48 h incubation at 37 °C, the cells were checked for the expression of GFP with the Fluorescence Microscope CKK41 (Olympus, Germany).

After successful rescue of viral progeny from the BAC, to remove the helper virus, the cells were harvested by scraping and spun at 4000 rpm and 4 °C for 10 min. The cell pellet was resuspended in 1 ml DMEM GlutaMAXX (Gibco/Thermo Scientific, USA) and viral particles were released from the cells by 3 times freeze-thawing. Cell debris was precipitated by 10 min centrifugation at 14,000 rpm. Two-hundred μl of the virus-containing supernatant in serial dilutions (10^− 1^–10^− 6^) was used to infect new DF-1 cells at confluency of 70–80%, in a 6-well plate. Three days later, cells from the most infected wells were harvested and blind passages were repeated until RFV was eradicated. This was tested by PCR using the RFV-specific primers listed in Table 1. Passages were continued to get rid of the plasmid backbone and GFP cassette from the viral genome. Finally, in positive recMVA-producing cells, the integrity of the gene inserts was checked by sequencing of the *Del*VI PCR product.

## Virus stock preparation by ultracentrifugation

DF-1 cells were seeded in T-75 flasks and then at confluency of 80–90% were infected with the recMVA viruses. Two days post-infection, the cells were harvested by scrapping; washed with 1 × PBS and then resuspended in 10 mM Tris pH 9. After 3 times freeze-thawing, the samples were sonicated 3 times on ice, each for 30 s, at 100% power. The cell debris was sedimented at 4000×*g*, 4 °C for 5 min. In UltraClear tubes (Bechman Coulter, Germany), the supernatant containing viral particles was added to 36% sucrose and viral particles were pelleted with ultracentrifugation at 13,500 rpm and 4 °C for 1.5 h. Virus pellets were air-dried and resuspended in 200 μl 10 mM Tris pH 9 for further analysis.

## Electron microscopy

Negative staining was carried out to visualize recMVA particles by electron microscopy (EM). For this, 5 μl of the purified virus suspension was put on a S162 flying copper grid. After 5 min, the copper grid was washed with 5 μl water and then were stained with 0.5% uranyl acetate in water for 20 s. The fluid film was removed with a paper and the samples were then analyzed by EM.

## Immunofluorescence staining for binding assay

In 12-well plates, UV-sterilized cover slips were coated with 1:20 diluted L-lysine in PBS at 37 °C for 1 h. After washing with 1 × PBS, DF-1 cells were seeded on the coated cover slips. At confluence of 70–80%, the culture medium was removed; the cells were washed twice with ice-cold PBS and then incubated with virus supernatant at 4 °C for 2–3 h. The cells were then washed (3x) carefully with PBS; fixed with 4% PFA for 20 min and then incubated for 30 min at 37 °C with anti-Vaccinia virus primary antibody (Acris Antibodies, Germany) diluted in PBS + 10% serum. Slides are washed twice, each for 5 min, with 1 × PBS before adding goat Alexa Fluor 594-conjugated anti-rabbit IgG (H + L) secondary antibody in PBS + 10% serum. After 30 min incubation at 37 °C in a dark chamber, the cover slips were washed (× 2, each for 5 min) and then transferred upside down to a glass slide with DAPI mounting solution. The next day, the cells were analyzed with the Confocal Microscope Fluoview FV10 (Olympus, Germany).

## Virus titration - plaque forming units

Virus titer was measured for three virus preparations. Briefly, ten-fold serial dilutions (10^− 4^–10^− 9^) of virus suspension in 2% FCS medium were plated out in duplicates on confluent DF-1 monolayers in 6-well plates. After 2 h infection at 37 °C, the inoculum was removed and the cells were washed with 1 × PBS followed by 2 days incubation at 37 °C in 2% FCS medium. After that, the medium was removed and the cells were fixed with a 1:1 mixture of ice-cold (− 20 °C) acetone:methanol for 5 min at RT. The cells were then blocked for 1 h at RT with PBS + 3% FCS and were incubated with rabbit anti-Vaccinia virus primary antibody (Acris Antibodies, Germany), diluted 1:2000 in PBS + 3% FCS, for 1 h at RT. After washing (× 3) with PBS + 3% FCS, the cells were incubated with peroxidase-conjugated anti-rabbit IgG (1:5000 in PBS + 3% FCS) for 1 h at RT with gentle rocking. The cells were washed (× 3) and then incubated with the True-Blue Peroxidase Substrate for about 10 min. Stained plaques were counted and virus titer was calculated in PFU/ml by multiplying the mean plaque number (big, clearly blue plaques, not including satellite plaques) by the magnitude of dilution.

## Data Availability

All data are fully available without restriction.

## Abbreviations

BAC: Bacterial Artificial Chromosome
CEF: Chicken Embryo Fibroblast
Del: Deletion
GFP: Green Fluorescence Protein
Kan: Kanamycin
MVA: Modified Vaccinia Virus Ankara
NoV: Norovirus
PCR: Polymerase Chain Reaction
PFU: Plaque Forming Unite
recMVA: recombinant MVA
RFP: Rabbit Fibroma Virus
WT: Wild Type
